# Modulation of tumor immune microenvironment by TAS-115, a multi-receptor tyrosine kinase inhibitor, promotes antitumor immunity and contributes anti-PD-1 antibody therapy

**DOI:** 10.1038/s41598-023-35985-w

**Published:** 2023-05-31

**Authors:** Toshihiro Shibutani, Risa Goto, Isao Miyazaki, Akihiro Hashimoto, Takamasa Suzuki, Keiji Ishida, Tomonori Haruma, Toshihiro Osada, Takafumi Harada, Hidenori Fujita, Shuichi Ohkubo

**Affiliations:** Discovery and Preclinical Research Division, Taiho Pharmaceutical Co., Ltd., Tsukuba, Ibaraki Japan

**Keywords:** Cancer immunotherapy, Cancer microenvironment

## Abstract

TAS-115 is an oral multi-receptor tyrosine kinase inhibitor that strongly inhibits kinases implicated in antitumor immunity, such as colony stimulating factor 1 receptor and vascular endothelial growth factor receptor. Because these kinases are associated with the modulation of immune pathways, we investigated the immunomodulatory activity of TAS-115. An in vitro cytokine assay revealed that TAS-115 upregulated interferon γ (IFNγ) and interleukin-2 secretion by T cells, suggesting that TAS-115 activated T cells. Gene expression analysis suggested that TAS-115 promoted M1 macrophage differentiation. In in vivo experiments, although TAS-115 exerted a moderate antitumor effect in the MC38 mouse colorectal cancer model under immunodeficient conditions, this effect was enhanced under immunocompetent conditions. Furthermore, combination of TAS-115 and anti-PD-1 antibody exhibited greater antitumor activity than either treatment alone. Flow cytometry analysis showed the increase in IFNγ- and granzyme B (Gzmb)-secreting tumor-infiltrating T cells by TAS-115 treatment. The combination treatment further increased the percentage of Gzmb^+^CD8^+^ T cells and decreased the percentage of macrophages compared with either treatment alone. These results highlight the potential therapeutic effect of TAS-115 in combination with PD-1 blockade, mediated via activation of antitumor immunity by TAS-115.

## Introduction

Therapy with blocking programmed cell death 1 (PD-1), PD-1 ligand 1 (PD-L1), and cytotoxic T lymphocyte antigen-4 (CTLA-4) becomes promising approach for cancer treatment^1–3^. Despite recent immunotherapies exhibiting dramatic benefits in cancer therapy, their efficacy is limited in certain populations due to the immunosuppressive tumor microenvironment (TME)^4–6^. To address this issue, combination of immunotherapies with immunomodulatory reagents is considered a useful therapeutic strategy. Indeed, lenvatinib and the anti-PD-1 antibody, pembrolizumab combination therapy showed effective antitumor activity; subsequently, it was approved for endometrial cancer^7^ and renal cell carcinoma (RCC)^8^.

TAS-115 is a small-molecule inhibitor that inhibits several kinases, including colony stimulating factor 1 receptor (CSF1R), vascular endothelial growth factor receptor (VEGFR), MET, and platelet-derived growth factor receptor (PDGFR). The half-maximal inhibitory concentration (IC_50_) values of TAS-115 against CSF1R, MET, VEGFR2, PDGFRα, and PDGFRβ enzymatic activities have been reported to be 15, 32, 30, 0.81, and 7.1 nmol/L, respectively. Additionally, TAS-115 treatment has been confirmed to inhibit downstream signals of these molecules in tumor cells^9–11^. TAS-115 has shown antitumor activity against tumors harboring MET abnormalities, osteosarcoma, and bone metastatic tumors. Mechanisms of its antitumor activity are considered via anti-angiogenesis and/or direct inhibition of kinase signals associated with tumor growth, including CSF1R, MET, and PDGFRs^9,10,12^. Currently, phase 3 clinical trial against osteosarcoma is ongoing (JapicCTI-205335).

CSF1R is an important molecule modulating macrophage, and CSF1R inhibition depletes macrophages in tumor^13,14^. Macrophages are a major component of TME, and mainly, tumor-infiltrating macrophages are divided into two types. One is immune activating phenotype, M1 macrophages and another is immunosuppressive phenotype, M2 macrophages, and the existence of these macrophages influences antitumor immunity^15^. Other reports showed that M2 macrophages are more dependent on CSF1R signal than M1 macrophages^16^.

VEGF is a key TME modulatory molecule secreted from tumor cells, cancer-associated fibroblasts, myeloid-derived suppressor cells (MDSCs), and macrophages; it suppresses antitumor immunity^6,17,18^. VEGF/VEGFR signal inhibition mediated anti-angiogenesis is considered an ideal approach for improving immunosuppressive TME, and several anti-angiogenesis reagents and immune checkpoint inhibitors (ICIs) have been approved as part of combination therapies for non-small cell lung cancer, hepatocellular carcinoma, and RCC^19–22^.

Therefore, dual inhibition of CSF1R and VEGFR might be expected to activate antitumor immunity better than single kinase inhibition, either CSF1R or VEGFR, by modulating TME^23–25^. The specific features of TAS-115 motivated us to investigate the immunomodulatory effects of TAS-115 and the combined effect of TAS-115 with ICIs.

In this study, we investigated the effects of TAS-115 on macrophages and T cell activation in vitro and in vivo. We additionally evaluated the in vivo antitumor efficacy and tumor-infiltrating immune cell population following combination of TAS-115 and anti-PD-1 antibody. Our data suggested that TAS-115 could boost the antitumor efficacy of anti-PD-1/PD-L1 antibodies by enhancing antitumor immunity.

## Results

### TAS-115 inhibits CSF1R signal in macrophages and modulates its phenotype

**Figure 1:**
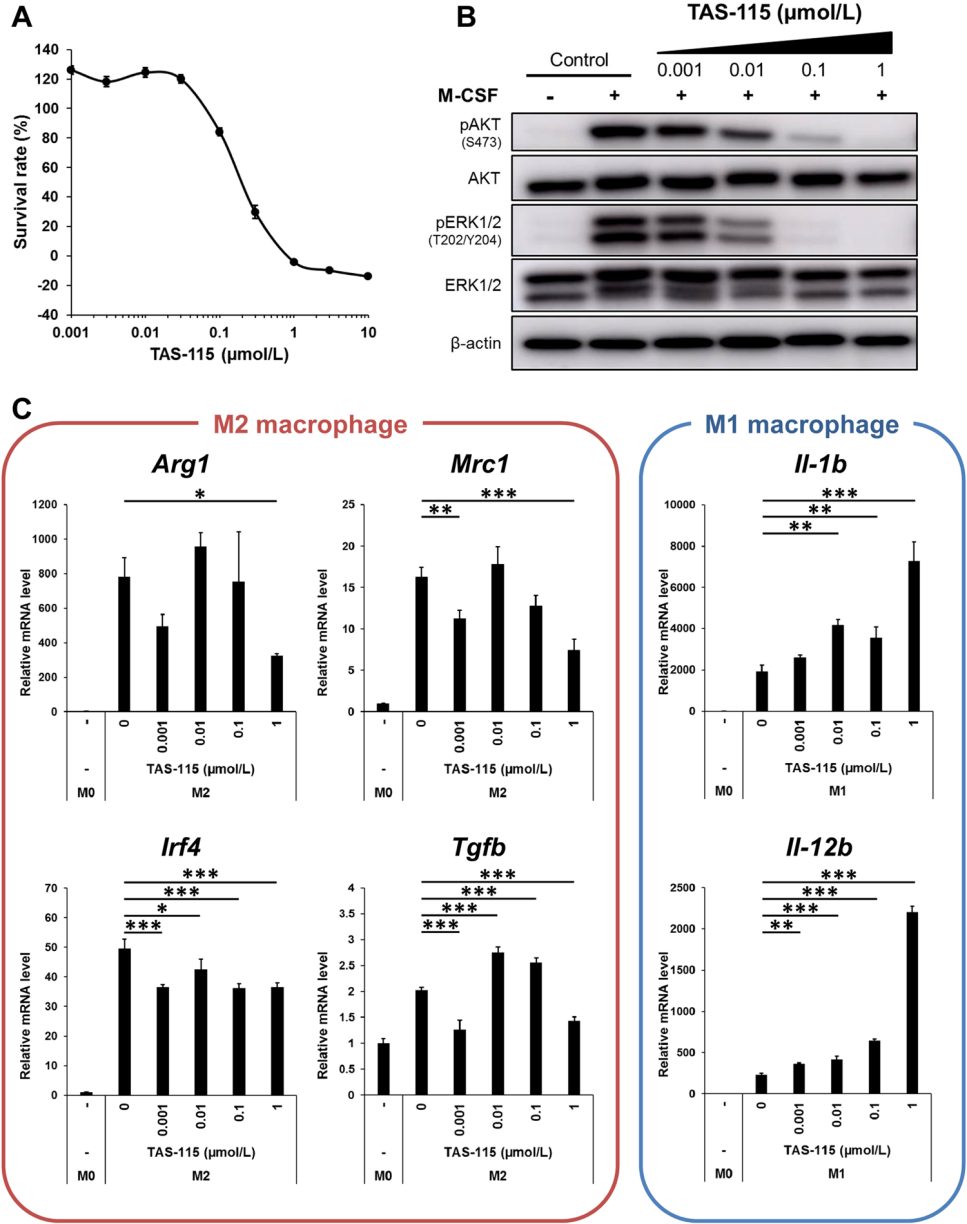
TAS-115 inhibits immune-suppressive macrophages. (**A**) The survival rate (%) of BMDMs treated with the indicated concentrations of TAS-115 for 4 days under M-CSF stimulation. Values indicate the ratio of TAS-115 treated versus DMSO control. Error bars indicate the S.D. (N = 3). (**B**) Western blot analysis of phospho-AKT and ERK in BMDMs stimulated with M-CSF for 5 min at the indicated concentrations of TAS-115. The original gel images are shown in Supplementary Fig. 6. (**C**) The mRNA expression levels of M2 macrophage markers (*Mrc1*, *Inf4*, *Arg1* and *Tgfb*) in IL-4 treatment-induced M2-polarized BMDMs and M1 macrophage markers (*IL-12b* and *IL-1b*) in both IFNγ and LPS treatment-induced M1-polarized BMDMs following treatment with TAS-115. M0 indicates BMDMs not treated with IL-4 or IFNγ and LPS. Error bars indicate the S.D. (N = 3). Statistical significance was determined by Dunnett test vs. 0 µmol/L TAS-115 (*p < 0.05, **p < 0.01, ***p < 0.001).

To address the immunomodulatory activity of TAS-115, we investigated whether TAS-115 could inhibit macrophage-colony stimulating factor (M-CSF)-dependent macrophage survival based on the inhibitory activity of TAS-115 against CSF1R signaling in bone marrow-derived macrophages (BMDMs). Mouse bone marrow cells were stimulated with M-CSF to differentiate into BMDMs under M-CSF stimulation and the generated BMDMs were maintained in culture medium containing M-CSF. TAS-115 inhibited cell survival of BMDMs with an IC_50_ value of 0.20 µmol/L (Fig. 1A). By western blot analysis, TAS-115 inhibited M-CSF-induced phospho-AKT and phospho-ERK in BMDMs at concentration more than 0.1 µmol/L (Fig. 1B). These results demonstrate that TAS-115 inhibits M-CSF-dependent macrophage survival.

Next, the effects of TAS-115 on M1 and M2 macrophage polarization were evaluated by mRNA quantification. BMDMs were treated with interleukin (IL) -4 alone or both interferon γ (IFNγ) and lipopolysaccharide (LPS) to induce M2 or M1 macrophages, respectively. To avoid the influence of inhibition on M-CSF dependent survival by TAS-115 treatment, this assay was performed in the absence of M-CSF. The differentiation to M2 or M1 macrophages was confirmed by RT-PCR analysis detecting upregulation of M2 macrophage-related gene (i.e., *Arg1*, *Mrc1*, *Irf4*, and *Tgfb*) expression in IL-4-treated BMDMs or M1 macrophage-related gene (i.e., *Il-1b* and *Il-12b*) expression in both IFNγ- and LPS-treated BMDMs compared with those in non-treated BMDMs (M0 macrophages). Concurrent treatment with a high dose of TAS-115 suppressed the M2 macrophage-related gene expression under an M2 polarizing condition. In contrast, the M1 macrophage-related gene expression was upregulated by TAS-115 at concentration more than 0.01 µmol/L under an M1 polarizing condition (Fig. 1C). These data indicate that TAS-115 promotes the differentiation of M1 macrophages.

### TAS-115 directly promotes T cell activation

**Figure 2:**
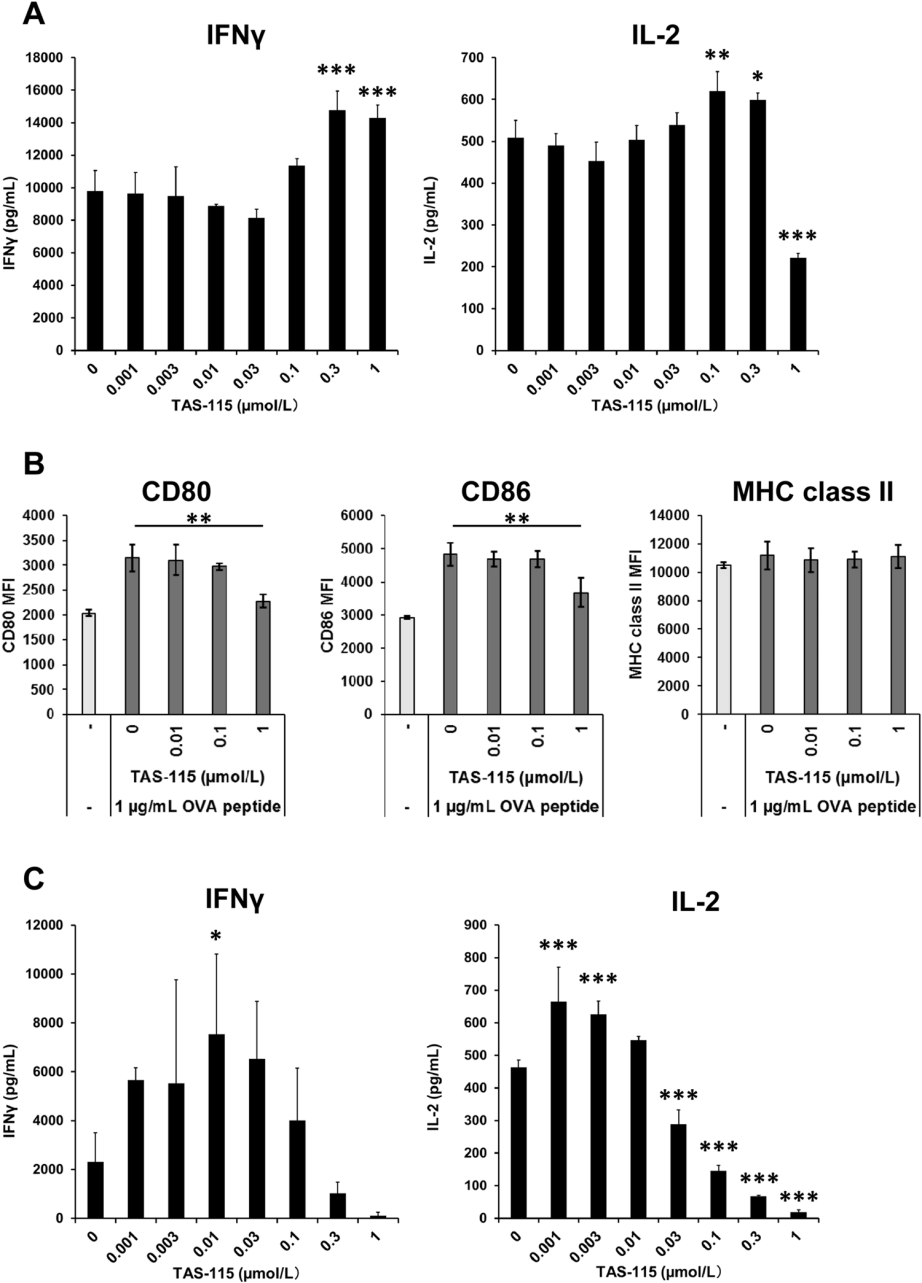
TAS-115 upregulated IFNγ and IL-2 secretion by T cells. (**A**) IFNγ and IL-2 secretion by OT-1 mouce splenocytes stimulated with OVA peptide under treatment with indicated concentrations of TAS-115. IFNγ and IL-2 levels were determined 2 days and 1 day after initiation of stimulation, respectively. Error bars indicate the S.D. (N = 3). (**B**) The expression levels of CD80, CD86, and MHC class II on CD11c-positive cells stimulated with OVA peptide and indicated concentration of TAS-115. Error bars indicate the S.D. (N = 3). (**C**) IFNγ and IL-2 secretion from hPBMCs stimulated with anti-CD3/CD28 cocktail and indicated concentrations of TAS-115 for 2 days. Statistical significance was determined by Dunnett test vs. 0 µmol/L TAS-115 (*p < 0.05, **p < 0.01, ***p < 0.001).

To investigate the effects of TAS-115 on T cells, we evaluated IFNγ and IL-2 secretion by splenocytes isolated from OT-1 mouse spleens. OT-1 splenocytes can be activated by ovalbumin (OVA) peptide, enabling the evaluation of the effects of inhibitors on antigen-specific T cell activation. Splenocytes were exposed to several concentrations of TAS-115 under OVA peptide stimulation, and the secreted IFNγ and IL-2 were evaluated. IFNγ secretion by OT-1 splenocytes was enhanced by 0.3 and 1 µmol/L TAS-115 and IL-2 secretion was enhanced by 0.1 and 0.3 µmol/L TAS-115. TAS-115 reduced IL-2 secretion at concentration of 1 µmol/L (Fig. 2A). In this assay system, increase in IFNγ and IL-2 secretion might be also observed in the case which TAS-115 promoted dendritic cell (DC) maturation. To exclude this possibility, the effects of TAS-115 on DC maturation were investigated. CD11c-positive cells, isolated from OT-1 mouse splenocytes, were cultured in a medium containing OVA peptide and TAS-115; subsequently, we evaluated the expression of DC maturation markers CD80, CD86, and MHC class II (I-A/I-E). However, the levels of these maturation markers were not enhanced by TAS-115 (Fig. 2B). Additionally, TAS-115 did not enhance the expression of these markers on CD11c-positive cells cultured in a medium containing LPS, used as DC maturation reagents (Supplementary Fig. 2). These results indicated that TAS-115 upregulated antigen-induced T cell activation but did not activate DCs.

Furthermore, to confirm that TAS-115 treatment activates human T cells, we investigated IFNγ and IL-2 secretion by human peripheral blood mononuclear cells (hPBMCs). hPBMCs were exposed to TAS-115 under agonistic anti-CD3 and CD28 antibodies stimulation condition; subsequently, the secreted IFNγ and IL-2 were evaluated. Consistent with the results from studies with mouse splenocytes, TAS-115 treatment increased the secretion of IFNγ and IL-2 by hPBMCs (Fig. 2C). The increase in both IFNγ and IL-2 secretion by hPBMCs occurred with TAS-115 concentrations that were lower than that used in the study with mouse splenocytes. But, IFNγ secretion tended to be decreased by TAS-115 at concentration more than 0.3 µmol/L and IL-2 secretion was decreased by TAS-115 at concentration more than 0.03 µmol/L. These data indicate that TAS-115 directly affect T cells and enhance its activation-induced IFNγ and IL-2 secretion at certain concentrations.

### TAS-115 activates tumor-infiltrating T cells and inhibits tumor growth in syngeneic mouse model

**Figure 3:**
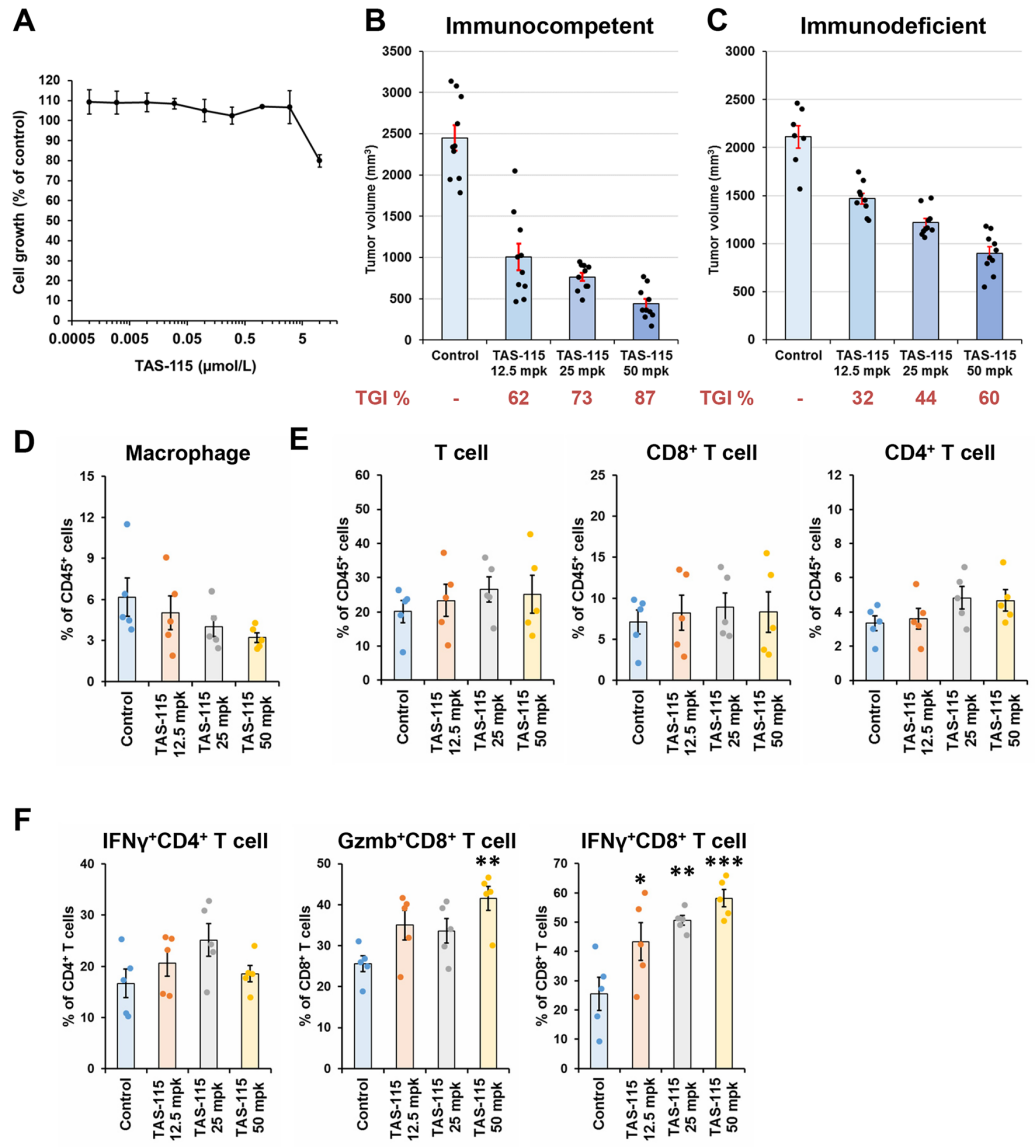
TAS-115 showed strong antitumor effect in immunocompetent condition and activated tumor-infiltrating CD8^+^ T cells. (**A**) The effects of TAS-115 against in vitro proliferation of MC38 cells. (**B**,**C**) Mean tumor volume values 21 days after initiation of TAS-115 treatment in MC38 tumor-bearing B6 mouse model (**B**) and SCID mouse model (**C**). The bar indicates the mean tumor volume in each group, and each error bar indicates the standard error (S.E.) (N = 7–10). Each plotted dot indicates the tumor volume of individual mice. Values under each graph indicate TGI% of mean tumor volume in each group. (**D**–**F**) Percentage of macrophage, T cell, and cytokine-secreting T cell populations in MC38 tumor on day 10. Macrophage populations are macrophages of CD45^+^ cells (**D**). T cell populations are CD8^+^ T cells and CD4^+^ T cells of CD45^+^ cells (**E**). Cytokine-secreting T cell populations are IFNγ^+^CD4^+^ T cells of CD4^+^ T cells, IFNγ^+^CD8^+^ T cells of CD8^+^ T cells, and Gzmb^+^CD8^+^ T cells of CD8^+^ T cells (**F**). The bar indicates the mean in each group, and each error bar indicates the S.E. (N = 5). Each plotted dot indicates the individual value. Statistical significance was determined by Dunnett’s test vs. control (*p < 0.05, **p < 0.01, ***p < 0.001).

Since TAS-115 promoted M1 macrophage differentiation and cytokine secretion by T cells in vitro, we hypothesized that TAS-115 could enhance antitumor immunity in vivo. To test this hypothesis, we investigated the in vivo antitumor efficacy of TAS-115 in a syngeneic MC38 colorectal cancer model. In the in vitro cell viability assay, TAS-115 did not show the potent cell growth inhibition against MC38 cells (Fig. 3A). Nevertheless, in the in vivo study, TAS-115 significantly inhibited tumor growth in a dose-dependent manner (TGI% values were 62%, 73%, and 87% at doses of 12.5, 25, and 50 mg/kg, respectively) (Fig. 3B). Interestingly, TAS-115 was less active against MC38 tumors in severe combined immunodeficiency (SCID) mice (TGI% values were 32%, 44%, and 60% at doses of 12.5, 25, and 50 mg/kg, respectively) (Fig. 3C). These results indicate that the antitumor efficacy of TAS-115 is partially mediated by adaptive immunity.

Next, we evaluated tumor-infiltrating immune cell populations in the MC38 syngeneic model. The tumors were collected after 9 days of daily administration of TAS-115, and tumor-infiltrating immune cells were analyzed by flow cytometry (Fig. 3D–F and Supplementary Fig. 3). In myeloid populations, macrophages tended to decrease in a TAS-115 dose-dependent manner at this time point (Fig. 3D). This macrophage reduction was significant at a later time point (Supplementary Fig. 4). TAS-115 treatment did not increase the percentage of CD8^+^ T cells and CD4^+^ T cells (Fig. 3E); however, TAS-115 treatment significantly increased IFNγ^+^CD8^+^ T cells and granzyme B (Gzmb)^+^CD8^+^ T cells in a dose-dependent manner (Fig. 3F). These results suggested that the decrease in macrophage population and the increase in IFNγ^+^ or Gzmb^+^CD8^+^ T cell population by TAS-115 contribute to the increased antitumor efficacy of TAS-115 in the MC38 syngeneic model.

### TAS-115 does not affect expression of immunomodulatory molecules on tumor cells

**Figure 4:**
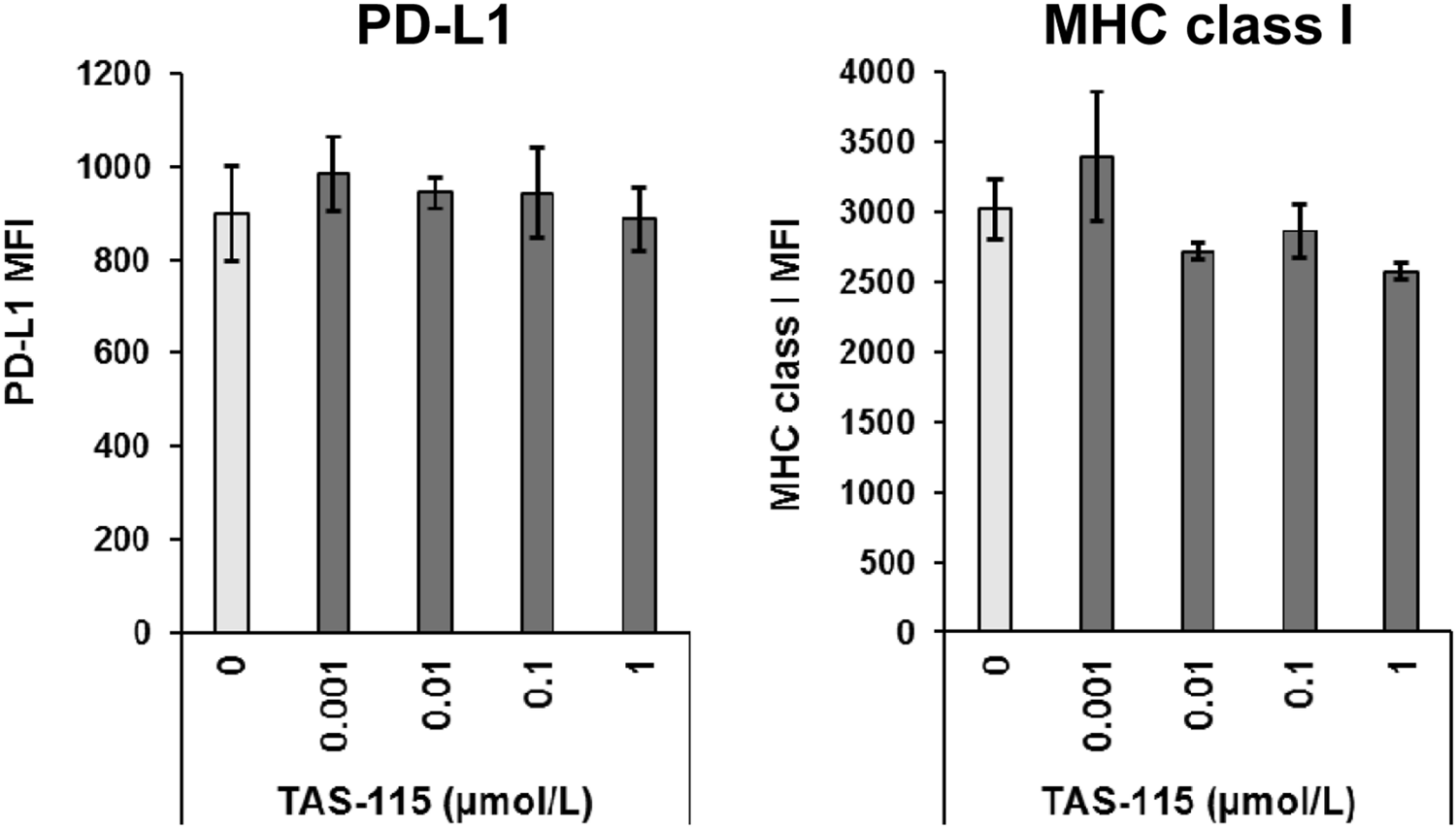
The expression levels of IFNγ-induced PD-L1 and MHC class I on MC38 cells treated with TAS-115. Indicated concentrations of TAS-115 represent the final concentrations used in this assay. Error bars indicate the S.D. (N = 3).

Some studies have suggested that the modulation of the expression of PD-L1 on tumor cells affects antitumor immunity and that MET signaling is associated with the expression of PD-L1 on tumor cells^26^. Because MET is a target of TAS-115, we evaluated PD-L1 expression on MC38 cells treated with TAS-115 by flow cytometry analysis. TAS-115 did not alter IFNγ-induced PD-L1 expression levels (Fig. 4). Another possible mechanism that activates antitumor immunity is the upregulation of antigen-MHC class I complex on tumor cell surface. Since this complex formation is dependent on MHC class I expression, we evaluated MHC class I (H2-K^b^) expression on MC38 cells under TAS-115 treatment. TAS-115 did not alter IFNγ-induced MHC class I expression levels of MC38 cells (Fig. 4). These data suggested that TAS-115 does not enhance antitumor immunity by affecting PD-L1 and MHC class I expression on tumor cells.

### Combined treatment of TAS-115 and anti-PD-1 antibody enhances antitumor immunity

**Figure 5:**
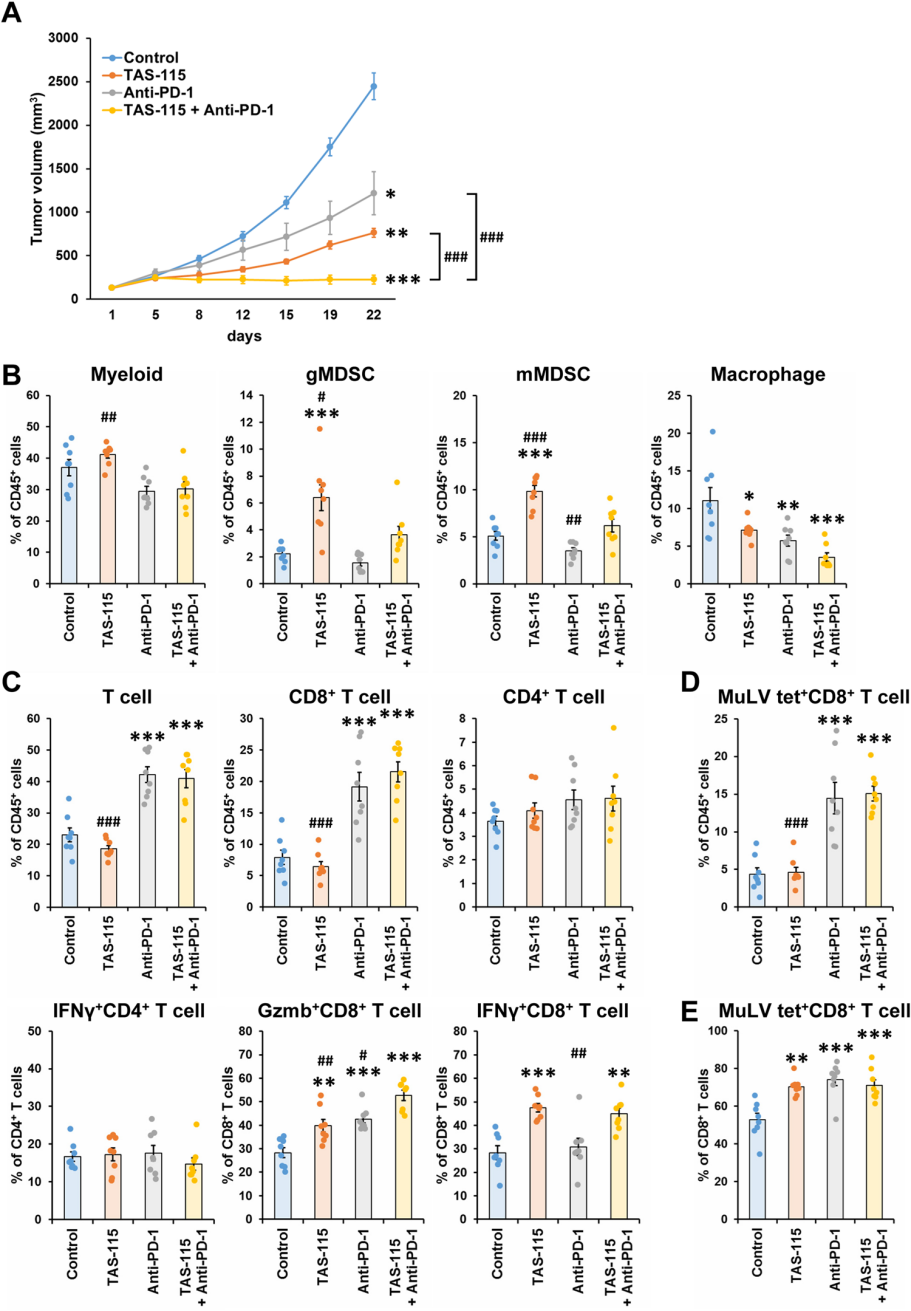
Combination of TAS-115 and anti-PD-1 antibody showed strong antitumor efficacy and activated cytotoxicity of tumor-infiltrating CD8^+^ T cells. (**A**) Mean tumor volume values of vehicle, TAS-115 (25 mg/kg), anti-PD-1 antibody (0.05 mg) or combination of TAS-115 and anti-PD-1 antibody in MC38 tumor-bearing B6 mouse model. Each plotted dot indicates the mean tumor volume, and each error bar indicates the S.E. (N = 10). (**B**–**E**) Percentage of myeloid, T cell, cytokine-secreting T cell and MuLV specific CD8^+^ T cell in MC38 tumor on day 10. Myeloid populations are total myeloid cells, gMDSCs, mMDSCs, and macrophages of CD45^+^ cells (**B**). T cell populations are total T cells, CD8^+^ T cells, and CD4^+^ T cells of CD45^+^ cells, and cytokine-secreting T cell populations are IFNγ^+^CD4^+^ T cells of CD4^+^ T cells, IFNγ^+^CD8^+^ T cells of CD8^+^ T cells and Gzmb^+^CD8^+^ T cells of CD8^+^ T cells (**C**). MuLV specific CD8^+^ T cell populations are MuLV specific CD8^+^ T cells of CD45^+^ cells (**D**) and MuLV specific CD8^+^ T cells of CD8^+^ T cells (**E**). The bar indicates the mean in each group, and each error bar indicates the S.E. (N = 8). Each plotted dot indicates the individual value. Statistical significance was determined by Tukey’s test vs. control (*p < 0.05, **p < 0.01, ***p < 0.001) and vs. TAS-115 + Anti-PD-1 (^#^p < 0.05, ^##^p < 0.01, ^###^p < 0.001).

Thus far, the data indicated that TAS-115 exhibited immunomodulatory effects and that the antitumor efficacy of TAS-115 was enhanced under immunocompetent conditions. Next, we examined the antitumor efficacy of combination of TAS-115 and anti-PD-1 antibody in the MC38 syngeneic model. Although single treatment with TAS-115 or anti-PD-1 antibody moderately suppressed tumor growth, combined treatment with TAS-115 and anti-PD-1 antibody exhibited greater tumor growth inhibition than either treatment alone (Fig. 5A). The combination treatment did not cause body weight loss during the study. These results suggested that the antitumor efficacy of anti-PD-1 antibody was enhanced by TAS-115.

We analyzed tumor-infiltrating immune cells to investigate the mechanisms underlying the enhanced antitumor activity of combination treatment with TAS-115 and anti-PD-1 antibody (Fig. 5B–E). The macrophage population was significantly decreased by single treatment with TAS-115 or anti-PD-1 antibody compared with the control treatment. In the combination treatment group, the reduction in the macrophage population was greater than either treatment alone. In other myeloid cells, although TAS-115 upregulated granulocytic MDSC (gMDSC) and monocytic MDSC (mMDSC) populations, these effects were weakened by combined with anti-PD-1 antibody (Fig. 5B). Both TAS-115 alone and anti-PD-1 antibody alone increased the Gzmb^+^CD8^+^ T cell population; importantly, the combination treatment further increased this population compared with the individual treatments. The IFNγ^+^CD8^+^ T cell population was increased by TAS-115 alone or in combination with anti-PD-1 antibody. In contrast to CD8^+^ T cells, the IFNγ^+^CD4^+^ T cell population did not change in any treatment group (Fig. 5C).

Furthermore, we investigated whether TAS-115 influenced antigen-specific cytotoxic T cell (CTL) induction. Because MuLV is a well-known antigen of MC38 cells, MuLV-specific CTLs in MC38 tumors were detected using fluorescent-labeled tetramers^27^. Single treatment with anti-PD-1 antibody, but not TAS-115, increased the percentage of antigen-specific CTLs relative to total lymphocytes compared with the control treatment (Fig. 5D). Interestingly, treatment with TAS-115 alone increased the percentage of antigen-specific CTLs relative to CD8^+^ T cells to the same extent as anti-PD-1 antibody treatment alone or combination treatment (Fig. 5E). These results indicate that TAS-115 could upregulate the percentage of tumor-reactive CTLs relative to CD8^+^ T cells. In summary, combination of TAS-115 and anti-PD-1 antibody might enhance tumor-infiltrating tumor-reactive CTLs and exhibit better antitumor activity than either treatment alone.

## Discussion

Macrophages present in tumor tissue produce immunosuppressive effects in the TME, and several drugs targeting macrophages have been developed^6^. M-CSF/CSF1R signaling is associated with the survival and development of macrophages; therefore, CSF1R inhibition could improve antitumor immunity^13,14^. Recently, combination therapies with CSF1R inhibitors and anti-PD-1/PD-L1 antibodies have been examined in clinical trials^6,28^. TAS-115, a novel multikinase inhibitor with a distinct target profile, has shown strong inhibitory activity against CSF1R. It has been reported to inhibit the M-CSF/CSF1R signaling pathway and M-CSF-dependent macrophage differentiation into osteoclasts^10,11,29^. In accordance with the previous report, TAS-115 inhibited M-CSF-induced phospho-AKT and phospho-ERK in BMDMs, subsequently affecting M-CSF dependent BMDM survival. Interestingly, TAS-115 inhibited M2 macrophages at high concentrations but promoted M1 macrophages at low concentrations. One possible explanation for the effects of TAS-115 on macrophages is the specific kinase inhibitory profile of TAS-115. First, compared with M1 macrophages, M2 macrophages have been reported to be more sensitive to M-CSF^16^, suggesting that CSF1R signal inhibition by TAS-115 may contribute to blocking M2 macrophage differentiation and promoting M1 macrophage differentiation. Second, TAM family kinases TYRO3, AXL, and MER play roles in promoting M2 macrophage differentiation and inhibiting M1 macrophage differentiation. Indeed, several TAM family kinase inhibitors have been reported to decrease the M2 macrophage population and increase M1 macrophage population in tumors^30,31^. TAS-115 showed significant inhibitory activity against TYRO3, AXL, and MER in the enzyme assay (Supplementary Table 1). Therefore, in addition to CSF1R inhibition, inhibition of TAM family kinases by TAS-115 might contribute to blocking M2 macrophage differentiation and promoting M1 macrophage differentiation, and these effects of TAS-115 could improve immune suppressive TME. Further analysis of tumor-infiltrating immune cells focusing on the detection of macrophage subtypes is required to clarify the effects of TAS-115 on macrophage differentiation in tumors. These findings show that TAS-115 has the potential to inhibit M2 macrophage differentiation and promote M1 macrophage differentiation in the TME, and these effects on macrophages could improve antitumor immunity.

IL-2 and IFNγ are associated with antitumor immunity of T cells^32^, and enhancement of either IL-2 or IFNγ secretion by T cells or splenocytes is expected to promote T cell-mediated antitumor efficacy. Because TAS-115 upregulated IL-2 and IFNγ secretion in stimulated mouse splenocytes and hPBMCs in vitro, it was suggested that TAS-115 promoted T cell activation. In mouse splenocytes, treatment with 1 µmol/L TAS-115 slightly decreased IL-2 secretion but did not inhibit IFNγ secretion, suggesting that 1 µmol/L TAS-115 did not directly damage mouse T cells. However, upregulation of IL-2 or IFNγ secretion by hPBMCs was observed at lower concentrations of TAS-115 than those observed in mouse splenocytes, and displayed a bell-shaped dose-response. Although the reason behind obtaining the bell-shaped dose-response in hPBMCs is unclear, it is suggested that human T cells are more sensitive to TAS-115 than mouse T cells, and species differences exist in the concentration promoting maximum secretion of IFNγ or IL-2 by TAS-115. As both IFNγ and IL-2 secretion by hPBMCs were decreased at concentrations above 0.3 µmol/L TAS-115, it may need to investigate the effects of high concentration of TAS-115 on human T cells. Consistent with these data, TAS-115 treatment increased IFNγ^+^CD8^+^ T cells in MC38 tumors. It was previously reported that DCs, but not T cells, express MET, and inhibition of MET signaling pathway promotes DC activation^33,34^. In addition to MET, VEGFR2 and CSF1R expression was detected in DCs (Supplementary Fig. 5). Because these molecules are targets of TAS-115, it is plausible that the inhibition of their signaling by TAS-115 treatment modulates the phenotypes of DCs and subsequently affects T cell activation. TAS-115 did not promote the expression of the DC maturation markers MHC class II, CD80, and CD86 under antigen or LPS stimulation (Fig. 2B and Supplementary Fig. 2), indicating that DCs were not activated by TAS-115 under our in vitro assay conditions. Consistent with these results, TAS-115 did not inhibit the downstream signalings of CSF1R and VEGFR2. Therefore, we concluded that TAS-115 exhibited potent effects by directly promoting T cell activation. However, the contribution of CSF1R and VEGFR2 to DCs remain unclear. The effects of TAS-115 on DCs may differ in the TME, where the ligands for these molecules are abundant.

Another specific feature of TAS-115 is its anti-angiogenic activity mediated by inhibition of VEGFR and MET^9^, suggesting the potential involvement of TAS-115 in antitumor immunity. The benefits of the combination treatment of anti-angiogenesis inhibitors such as bevacizumab, axitinib, and lenvatinib and anti-PD-1/PD-L1 antibodies have been reported in pre-clinical and even in clinical settings, and some combination therapies have already been approved as cancer treatments^19^. VEGF not only directly inhibits T cell function^23,35^ but also works as major factor promoting the formation of tumor vessels, which induces Fas-FasL interaction-mediated T cell apoptosis^36^. Additionally, MET expression is induced by inhibition of VEGF/VEGFR signaling, and MET signaling promotes angiogenesis in VEGFR inhibitor-resistant tumors, implying that dual inhibition of MET and VEGFR coordinately inhibits tumor angiogenesis^37,38^. Therefore, the antitumor effect of TAS-115 on MC38 in an immunodeficient mouse model may be due to its anti-angiogenic activity through the dual inhibition of MET/VEGER. Additionally, the inhibition of macrophages by TAS-115 is expected to decrease VEGF in the TME because macrophages are a source of VEGF^35^. Thus, it is plausible that TAS-115 might exert antitumor immunity by inhibiting angiogenesis more potently through two actions: direct inhibition of MET and VEGFR and indirect inhibition of VEGF production by macrophages.

To clarify whether the immunomodulatory activity of TAS-115 contribute to its antitumor activity, we compared the antitumor efficacy of TAS-115 against MC38 tumors in immunocompetent and immunodeficient mice. TAS-115 exhibited more potent antitumor efficacy in an immunocompetent background, implying the importance of the adaptive immunity for its antitumor activity. In MC38 tumors, TAS-115 did not increase the tumor-infiltrating CD8^+^ T cells population, but increased IFNγ^+^CD8^+^ T cells and Gzmb^+^CD8^+^ T cells. Since increasing IFNγ^+^ or Gzmb^+^CD8^+^ T cells is reported to be associated with antitumor immunity^39^, the antitumor efficacy of TAS-115 may be partially mediated by activated T cells. TAS-115 monotherapy showed greater antitumor efficacy than anti-PD-1 antibody monotherapy; however, the CD8^+^ T cell ratio in tumors was low. TAS-115 is expected to exhibit both anti-angiogenesis activity and increase the percentage of active T cell populations, Gzmb^+^CD8^+^ T cells, IFNγ^+^CD8^+^ T cells, and MuLV^+^CD8^+^ T cells, relative to CD8^+^ T cells in tumor. These effects may contribute to the superior efficacy of TAS-115 against anti-PD-1 antibody. TAS-115 monotherapy did not increase in IFNγ^+^CD4^+^ T cells relative to CD4^+^ T cells, indicating that TAS-115 showed the antitumor efficacy against MC38 tumors without enhancing CD4^+^ T cell function.

Furthermore, combination of TAS-115 and anti-PD-1 antibody showed greater antitumor efficacy than either treatment alone in the mouse syngeneic model. Combined treatment resulted in a more significant increase in granzyme B-secreting CD8^+^ T cells and a decrease in macrophages than either individual treatment, suggesting that these additional effects might enhance antitumor efficacy. In addition, combination with an anti-PD-1 antibody suppressed the negative effects of TAS-115 on MDSCs. The inhibition of CSF1R and VEGFR2 reportedly increases gMDSC and mMDSC, respectively^40,41^. However, anti-PD-1 antibody decreased these MDSC populations^42^. Therefore, the suppression of the increase in MDSC populations by anti-PD-1 antibody may partially contribute to the activation of antitumor immunity by the combination treatment.

We considered upregulation of IFNγ^+^CD8^+^ T cells by TAS-115 contributed to increase antigen-specific T cells in CD8^+^ T cells and enhance the antitumor efficacy of anti-PD-1 antibody. It has been reported that IFNγ secreted from T cells induces MHC class I expression on tumor cells and upregulates antigen presentation on tumor cells in the TME^43^; in turn, T cell expansion occurs through recognition of the antigen-MHC class I complex on tumor cells. IFNγ secreted from T cells also induces PD-L1 expression on tumor cells^44,45^. However, combined treatment with TAS-115 and anti-PD-1 antibody could suppress this negative aspect of upregulation of IFNγ^+^CD8^+^ T cells by TAS-115. Indeed, although VEGFR inhibitors induce the expression of PD-L1 on tumor cells, combination of anti-PD-1/PD-L1 antibody and VEGFR inhibitor exhibited greater antitumor activity than either treatment alone^24^. Additionally, loss of IFNγ signaling via JAK1 or JAK2 loss-of-function mutation is one of mechanisms of resistance to anti-PD-1/PD-L1 antibody treatment, implying the importance of IFNγ for antitumor immunity^46–48^. In summary, it is possible that induction of IFNγ^+^CD8^+^ T cells by TAS-115 results in an increase in antigen-specific CTLs and combination efficacy. However, we cannot rule out the possibility that the involvement of macrophages or other component cells in the TME in the increase in IFNγ^+^CD8^+^ T cells in tumors.

**Figure 6:**
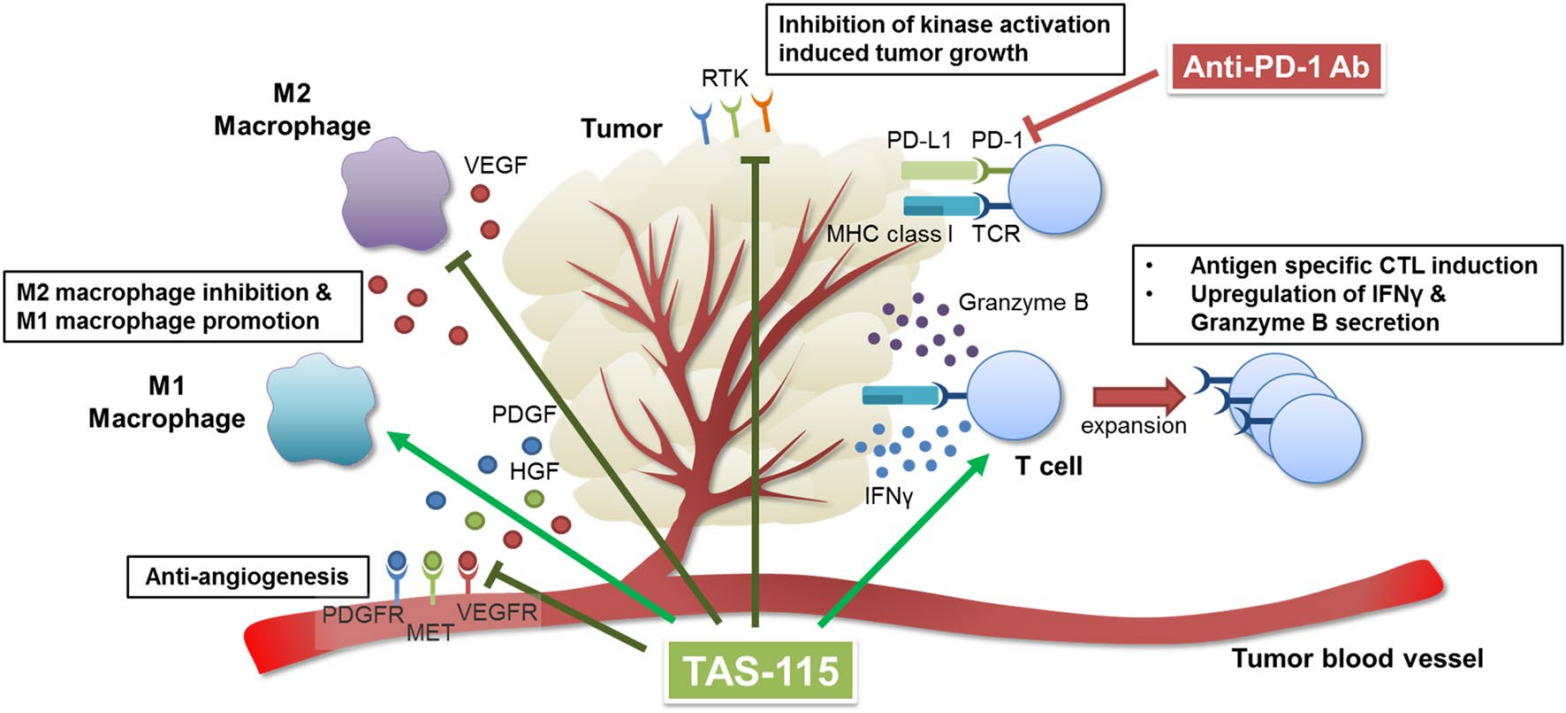
Graphical summary of putative effects of TAS-115 in tumor microenvironment. Multikinase inhibition of TAS-115 influences macrophage phenotype and function, T cell activation and function, and tumor growth.

Although the combination treatment of multi-receptor tyrosine kinase inhibitors with PD-1 blockade has shown potent antitumor effects, the effect is likely to be different for each inhibitor because of the variation in the inhibitory effects of kinases. For example, since lenvatinib strongly inhibits VEGF and fibroblast growth factor receptor (FGFR), VEGFR and FGFR dual inhibition would contribute to increasing antitumor immunity by reduction of the tumor-associated macrophages and anti-angiogenesis^39^. In contrast, TAS-115 has a unique kinase inhibitory profile that is involved in immunomodulation, such as CSF1R and VEGFR. Further investigations are needed to confirm the levels of contribution of individual kinase inhibition by TAS-115 on activation of antitumor immunity. We have shown that TAS-115 activated antitumor immunity via unique mechanisms (Fig. 6), activating T cells and regulating macrophage phenotypes probably mainly through CSF1R and VEGFR inhibition. These findings indicate novel immunomodulatory effects of TAS-115 and its potential efficacy in combination with ICIs, especially in patients exhibiting insufficient response to ICI alone.

## Methods

### Mice, cell lines and reagent

C57BL/6NCrl mice (B6 mice) and CB17/Icr-Prkdc[scid]/CrlCrlj mice (SCID mice) were obtained from CHARLES RIVER LABORATORIES JAPAN, INC. (Kanagawa, Japan). C57BL/6-Tg(TcraTcrb)1100Mjb/J mice (OT-1 mice), which express a transgenic TCR that is specific for OVA_257–264_ (SIINFEKL) peptide (OVA peptide) bound to H-2K^b^ were obtained from The Jackson Laboratory (Bar Harbor, ME), and were bred and maintained in Taiho pharmaceutical Co., Ltd. (Tokyo, Japan). All animal experiments were approval by the Institutional Animal Care and Use Committee at Taiho Pharmaceutical Co., Ltd. and were performed in accordance with guidelines of Taiho Pharmaceutical Co., Ltd.. All animal procedures were compliance with ARRIVE guidelines. MC38 cells were provided by Toyama University. TAS-115 [4-[2-fluoro-4-[[[(2-phenylacetyl)amino]thioxomethyl]amino]-phenoxy]-7-methoxy-N-methyl-6-quinolinecarboxamide] was prepared by Taiho Pharmaceutical Co., Ltd.. Anti-PD-1 antibody (clone RMP1-14) was purchased from Thermo Fisher Scientific (Waltham, MA).

### In vivo mouse model

Suspensions of 1 × 10^6^ MC38 cells were implanted subcutaneously into the right abdomen of male mice. When the mean tumor volume (TV) reached 80–200 mm^3^, tumor-bearing mice were randomly allocated into groups on the basis of their TVs, and treatments were initiated. TAS-115 (12.5, 25, or 50 mg/kg) was administered orally once daily. Anti-PD-1 antibody was dosed intraperitoneally on the initial day and day 8. For analysis of tumor-infiltrating immune cells, anti-PD-1 antibody was dosed only initial day. TV and body weight were measured twice per week. TV was calculated as follows: TV = [long (mm^2^)] × [short (mm^2^)]^2^/2, where [long] is the long diameter and [short] is the short diameter of the tumor. Tumor growth inhibition (TGI, %) was calculated as follows: 100 × {1 − [(TV_final_ − TV_initial_ for the treatment group)]/[(TV_final_ − TV_initial_ for the control group)]}. TV_initial_ and TV_final_ were the TV on the allocation day and final assessment day, respectively.

### Cytokine assay

Plated mouse splenocytes (2 × 10^5^ cells/well) were exposed to TAS-115 1 h before stimulation with OVA peptide (1 µg/mL) in a complete medium (RPMI-1640 supplemented with 10% heat-inactivated fetal bovine serum, 1% penicillin and streptomycin and 2-mercaptoethanol) and incubated for 1 or 2 days. The concentrations of IFNγ and IL-2 in culture supernatants were determined using Duo Set ELISA kit (R&D Systems Inc. Minneapolis, MN). ELISA was performed according to the kit protocol. For evaluation of IFNγ and IL-2 secretion from hPBMCs, cryopreserved hPBMCs were plated (2 × 10^5^ cells/well) and stimulated with anti-CD3 antibody and anti-CD28 antibody cocktail. Cryopreserved hPBMCs were obtained from Cellular Technology Limited (Shaker Heights, OH).

### Generation of BMDMs and M2 or M1 macrophage polarization

Bone marrow cells, collected from the femurs of mice, plated in a complete medium containing M-CSF (20 ng/mL). After 7 days of culture, BMDMs were harvested and used for further experiments. For M2 or M1 macrophage polarization, BMDMs were further cultured in a complete medium containing IL-4 (20 ng/mL) or both IFNγ (200 ng/mL) and LPS (400 ng/mL), respectively, in the absence of M-CSF.

### Cell signaling detection

BMDMs were lysed using Cell Extraction Buffer (Invitrogen, Waltham, MA), and the lysates were analyzed by western blot with antibodies against AKT (Cell Signaling Technology; CST, Danvers, MA), pAKT (CST), ERK (CST), pERK (CST), and β-actin (Abcam, Cambridge, UK).

### RT-qPCR analysis

BMDMs were harvested and seeded in 12-well plates. The cells were further cultured overnight under M2 or M1 macrophage polarization condition with or without TAS-115 treatment. Treated cells were harvested, and mRNA was extracted using the RNeasy mini kit (QIAGEN, Hilden, Germany). cDNA was synthesized using SuperScript IV VILO Master Mix (Invitrogen). Reaction mixture was prepared by mixing synthesized cDNA, TaqMan Fast Advanced Master Mix (Applied Biosystems, Waltham, MA), and commercially available pre-designed TaqMan probe. Gene expression was measured using the Vii^TM^7 (Applied Biosystems). Gene expression levels were determined using the ΔΔCt method.

### Quantification of cell survival and proliferation

BMDMs were treated with TAS-115 in a complete medium containing M-CSF. After 4 days, cell survival was evaluated using CellTiter-Glo^®^ (Promega, Madison, WI). MC38 cells were plated in a 96-well plate and cultured overnight. Subsequently, TAS-115 was added, and the cells were incubated for 3 days. Cellular proliferation was evaluated using CellTiter-Glo^®^.

### Flow cytometry analysis

For analysis of tumor-infiltrating immune cells, single cell suspensions were prepared from tumors using tumor dissociation kit (Miltenyi Biotec, Bergisch Gladbach, Germany). The cells were magnetically isolated using a CD45 isolation kit (Miltenyi Biotec) from suspension, and the isolated cells were stained with fluorochrome-labeled antibodies against CD45 (Thermo Fisher Scientific), CD90.2 (Thermo Fisher Scientific), CD8 (Thermo Fisher Scientific), CD4 (Thermo Fisher Scientific), CD11b (Thermo Fisher Scientific), Ly6G (Miltenyi Biotec), Ly6C (Miltenyi Biotec), and F4/80 (Miltenyi Biotec). For analysis of MC38-reactive T cells, the cells were stained with fluorochrome-labeled MuLV tetramer (MBL, Tokyo, Japan). The gating strategy is presented in Supplementary Fig. 1. For analysis of cytokine secretion in tumor-infiltrating immune cells, isolated cells were plated and cultured in a medium containing Cell stimulation cocktail (Thermo Fisher Scientific) and Protein transport inhibitor, Golgi STOP (BD, Franklin Lakes, NJ) for 4 h. Cultured cells were collected and stained with fluorochrome-labeled antibodies against CD45, CD90.2, CD8, and CD4. Subsequently, the cells were fixed and permeabilized using the Foxp3/Transcription permeabilization kit (Thermo Fisher Scientific) and stained with fluorochrome-labeled antibodies against IFNγ (Biolegend, San Diego, CA) and Granzyme B (Biolegend).

For analysis of cell surface protein expression on CD11c-positive cells and MC38 cells, CD11c-positive cells, isolated from C57BL/6Jj OT-1 mice using CD11c MicroBeads UltraPure, mouse (Miltenyi Biotec), were stained with fluorochrome-labeled antibodies against I-A/I-E (Thermo Fisher Scientific), CD80 (Thermo Fisher Scientific), and CD86 (Thermo Fisher Scientific). Plated MC38 cells were exposed to TAS-115 1 day before stimulation with IFNγ (50 ng/mL) and incubated overnight. The treated cells were harvested and stained with fluorochrome-labeled antibodies against PD-L1 (Thermo Fisher Scientific) and H2-K^b^ (Thermo Fisher Scientific).

Cell populations or mean fluorescence intensity (MFI) were evaluated using FACS verse (BD) and analyzed using the FlowJo software (BD).

### Statistical analysis

Statistical significance was analyzed using the Dunnett’s test and Tukey’s test with SAS version 9.2 (SAS Institute Japan, Tokyo, Japan). Statistical significance was set at p < 0.05.

### Supplementary Information

Supplementary Information.

## Data Availability

The datasets generated during and/or analyzed during the current study are available from the corresponding author on reasonable request.
