# Author Correction: Modulation of tumor immune microenvironment by TAS-115, a multi-receptor tyrosine kinase inhibitor, promotes antitumor immunity and contributes anti-PD-1 antibody therapy

**DOI:** 10.1038/s41598-024-55488-6

**Published:** 2024-02-27

**Authors:** Toshihiro Shibutani, Risa Goto, Isao Miyazaki, Akihiro Hashimoto, Takamasa Suzuki, Keiji Ishida, Tomonori Haruma, Toshihiro Osada, Takafumi Harada, Hidenori Fujita, Shuichi Ohkubo

**Affiliations:** Discovery and Preclinical Research Division, Taiho Pharmaceutical Co., Ltd., Tsukuba, Ibaraki Japan

Correction to: *Scientific Reports* 10.1038/s41598-023-35985-w, published online 31 May 2023

The original version of this Article contained an error in Figure 3E, where the labels of the middle graph and the right graph were interchanged. The correct label for the middle graph is “CD8^+^ T cell” and the correct label for the right graph is “CD4^+^ T cell”.

The original Figure 3 and accompanying legend appear below.

**Figure 3:**
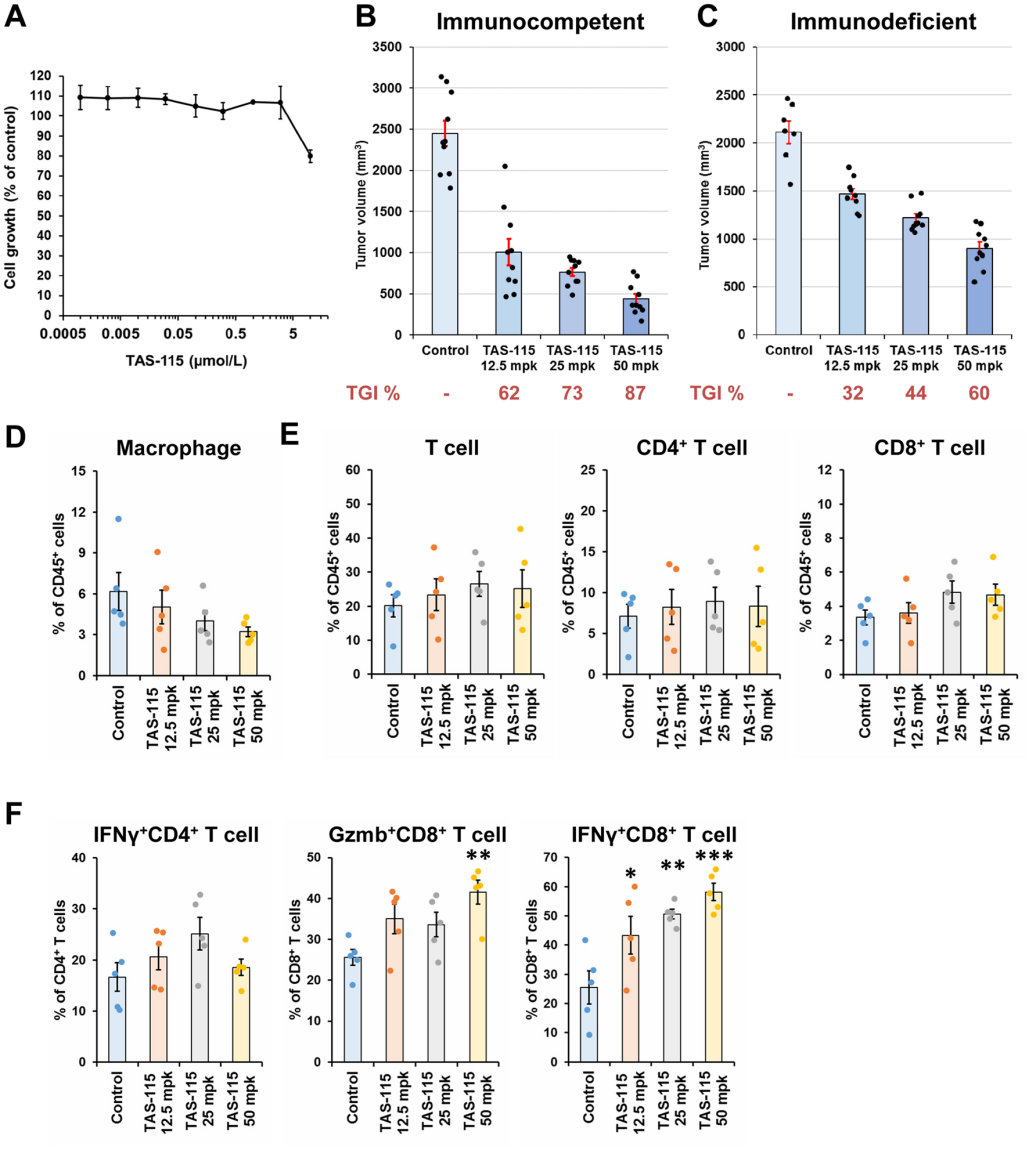
TAS-115 showed strong antitumor effect in immunocompetent condition and activated tumor-infiltrating CD8^+^ T cells. (**A**) The effects of TAS-115 against in vitro proliferation of MC38 cells. (**B**, **C**) Mean tumor volume values 21 days after initiation of TAS-115 treatment in MC38 tumor-bearing B6 mouse model (**B**) and SCID mouse model (**C**). The bar indicates the mean tumor volume in each group, and each error bar indicates the standard error (S.E.) (N = 7–10). Each plotted dot indicates the tumor volume of individual mice. Values under each graph indicate TGI% of mean tumor volume in each group. (**D**–**F**) Percentage of macrophage, T cell, and cytokine-secreting T cell populations in MC38 tumor on day 10. Macrophage populations are macrophages of CD45^+^ cells (**D**). T cell populations are CD8^+^ T cells and CD4^+^ T cells of CD45^+^ cells (**E**). Cytokine-secreting T cell populations are IFNγ^+^CD4^+^ T cells of CD4^+^ T cells, IFNγ^+^CD8^+^ T cells of CD8^+^ T cells, and Gzmb^+^CD8^+^ T cells of CD8^+^ T cells (**F**). The bar indicates the mean in each group, and each error bar indicates the S.E. (N = 5). Each plotted dot indicates the individual value. Statistical significance was determined by Dunnett’s test vs. control (*p < 0.05, **p < 0.01, ***p < 0.001).

The original Article has been corrected.

