# Supplementary material for: Modulation of tumor immune microenvironment by TAS-115, a multi-receptor tyrosine kinase inhibitor, promotes antitumor immunity and contributes anti-PD-1 antibody therapy
Source: Sci Rep. 2023 May 31;13:8821. doi: 10.1038/s41598-023-35985-w (PMC10232527; doi:10.1038/s41598-023-35985-w)
Supplement: Supplementary file 1 — Supplementary Information. [file 41598_2023_35985_MOESM1_ESM.pdf]

Modulation of tumor immune microenvironment by TAS-115, a multi-receptor tyrosine kinase inhibitor, promotes antitumor immunity and contributes anti-PD-1 antibody therapy

Toshihiro Shibutani[1,*], Risa Goto[1], Isao Miyazaki[1], Akihiro Hashimoto[1], Takamasa Suzuki[1], Keiji Ishida[1], Tomonori Haruma[1], Toshihiro Osada[1], Takafumi Harada[1], Hidenori Fujita[1], and Shuichi Ohkubo[1]

[1]Discovery and Preclinical Research Division, Taiho Pharmaceutical Co., Ltd., Tsukuba, Ibaraki, Japan.

[*]t-shibutani@taiho.co.jp

Supplementary Table 1. The half-maximal inhibitory concentration (IC_{50}) values of TAS-115 against TYRO3, AXL, and MER enzymatic activities.

Kinase	IC_{50} (nmol/L)
TYRO3	30.6
AXL	2.00
MER	4.16

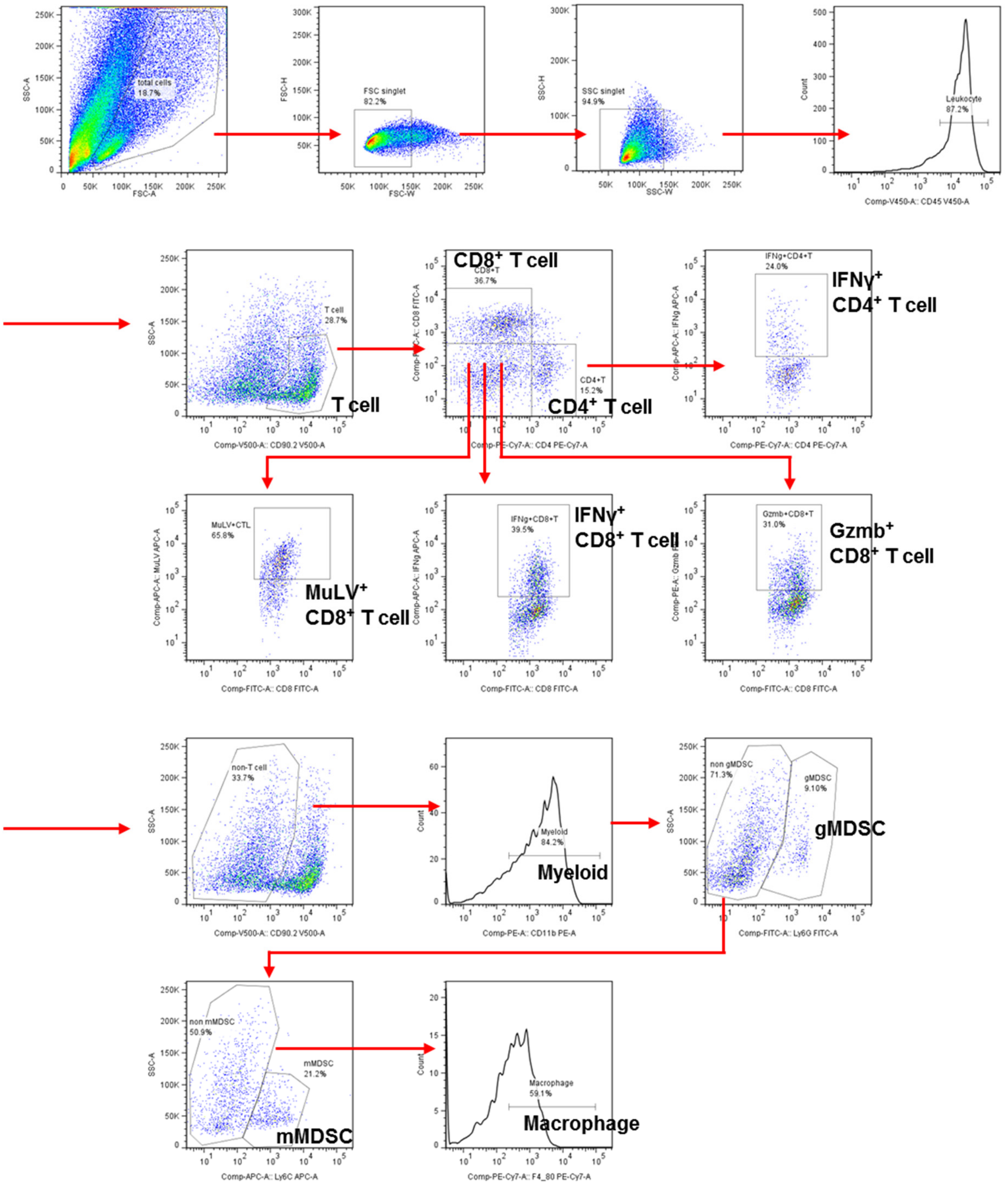

Supplementary Figure 1. Gating strategy of T cell (CD45$^+$CD90.2$^+$), CD8$^+$ T cell (CD45$^+$CD90.2$^+$CD8$^+$CD4$^-$), MuLV tet$^+$CD8$^+$ T cell (CD45$^+$CD90.2$^+$CD8$^+$CD4$^-$MuLV$^+$), IFNγ$^+$CD8$^+$ T cell (CD45$^+$CD90.2$^+$CD8$^+$CD4$^-$IFNγ$^+$), Gzmb$^+$CD8$^+$ T cell (CD45$^+$CD90.2$^+$CD8$^+$CD4$^-$Gzmb$^+$), CD4$^+$ T cell (CD45$^+$CD90.2$^+$CD8$^-$CD4$^+$), IFNγ$^+$CD4$^+$ T cell (CD45$^+$CD90.2$^+$CD8$^-$CD4$^+$IFNγ$^+$), Myeloid (CD45$^+$CD90.2$^-$CD11b$^+$), gMDSC (CD45$^+$CD90.2$^-$CD11b$^+$Ly6G$^+$), mMDSC (CD45$^+$CD90.2$^-$CD11b$^+$Ly6G$^-$Ly6C$^+$), and Macrophage (CD45$^+$CD90.2$^-$CD11b$^+$Ly6G$^-$Ly6C$^-$F4/80$^+$) for the flow cytometry analysis. Tumor-infiltrating immune cell populations were analyzed in indicated gates.

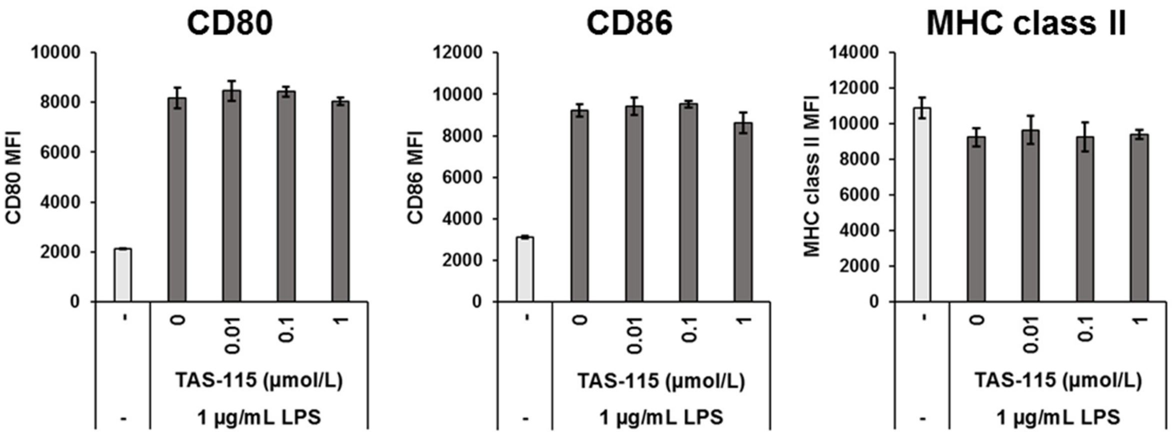

Supplementary Figure 2. The expression levels of CD80, CD86, and MHC class II on CD11c-positive cells stimulated with LPS and indicated concentration of TAS-115.

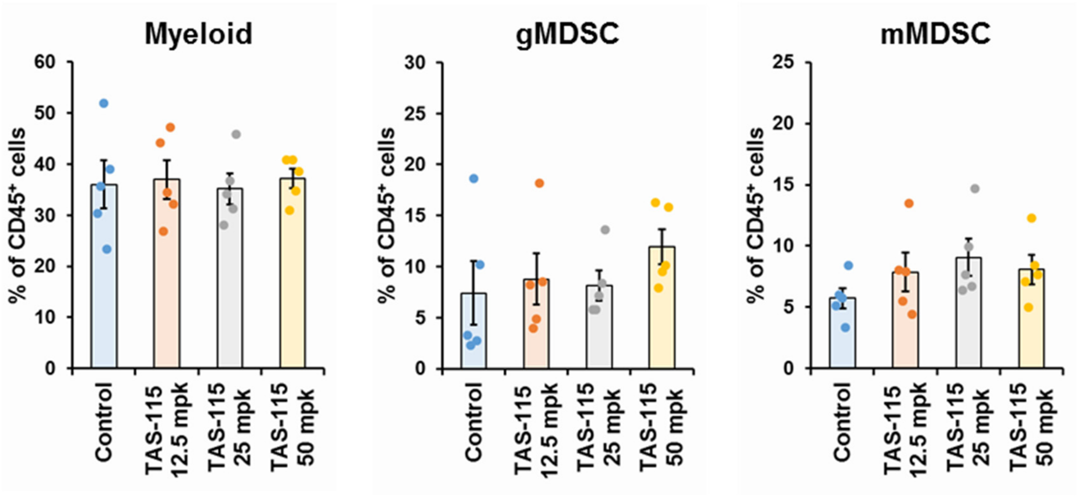

Supplementary Figure 3. Percentage of total myeloid cells, gMDSCs, and mMDSCs of CD45$^+$ cells in MC38 tumor on day 10.

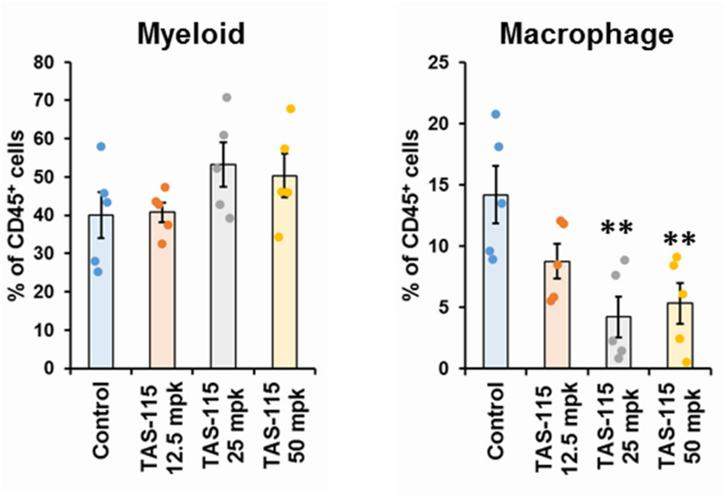

Supplementary Figure 4. Percentage of total myeloid cells and macrophages of $CD45^+$ cells in MC38 tumor on day 15. Statistical significance was determined by Dunnett's test vs. control (*p<0.05, **p<0.01)

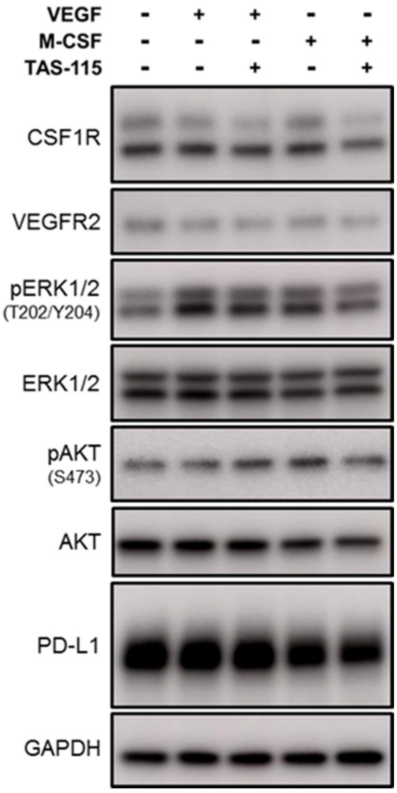

Supplementary Figure 5. Western blot analysis of CSF1R, VEGFR2, PD-L1, and downstream signals in CD11c-positive cells. The cells were stimulated with M-CSF or VEGF for 5 min at 0.1 μmol/L TAS-115. The original gel images are shown in Supplementary Figure 6.

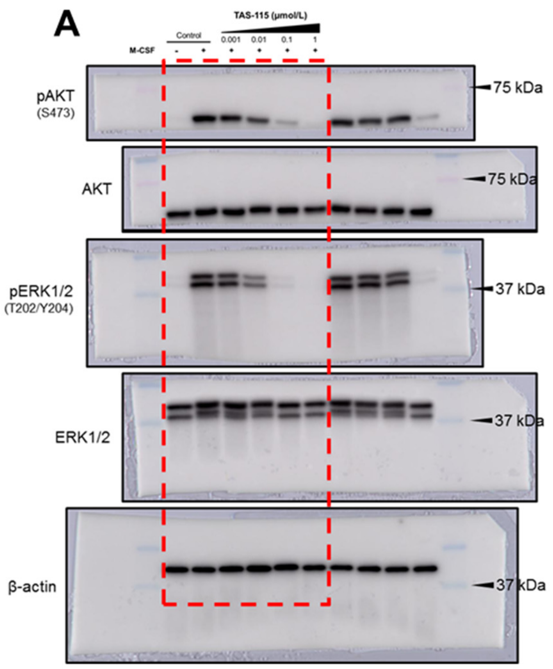

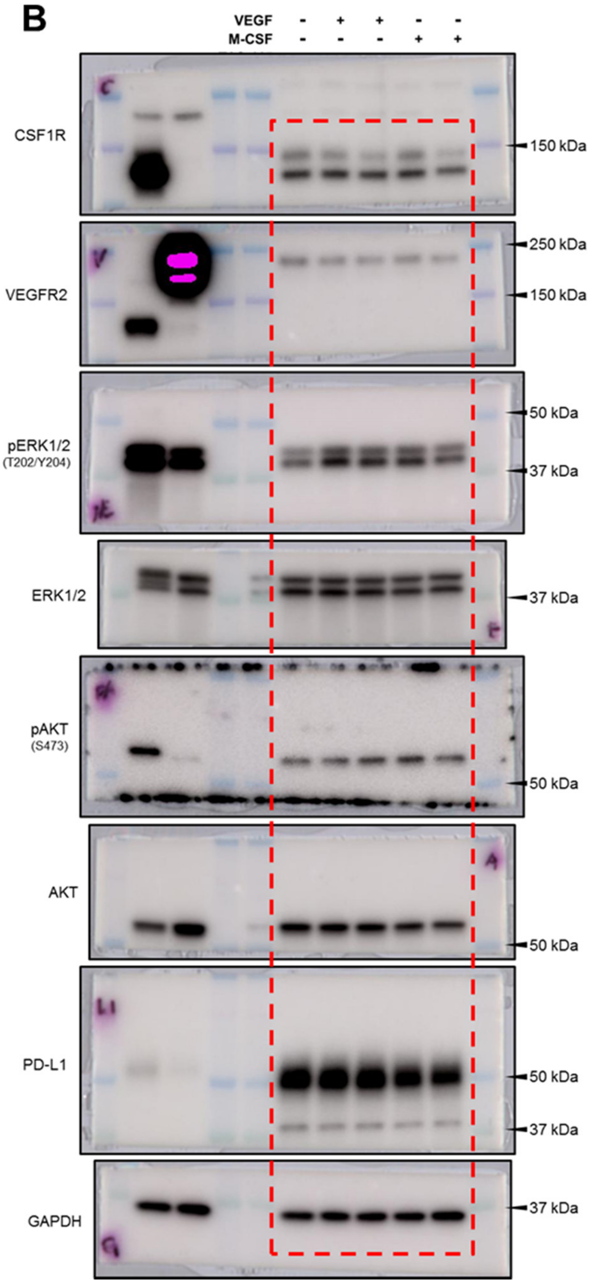

Supplementary Figure 6. Uncropped gel images of the western blot. The red dotted rectangle indicates the areas shown in (A) Figure 1B and (B) Supplementary Figure 5.

Supplementary Information

Enzymatic analysis

Half-maximal inhibitory concentration values of TAS-115 against TYRO3, AXL, and MER enzymatic activities were determined by mobility shift assay (Carna Biosciences, Inc., Hyogo, Japan).

Detection of protein expression and cell signaling in CD11c$^+$ cells

CD11c$^+$ cells were lysed using M-PER™ Mammalian Protein Extraction Reagent (Thermo Fisher Scientific, Waltham, MA), and the lysates were analyzed by western blot with antibodies against CSF1R (Cell Signaling Technology; CST, Danvers, MA), VEGFR2 (CST), AKT (CST), pAKT (CST), ERK (CST), pERK (CST), PD-L1 (Abcam, Cambridge, UK), and GAPDH (Trevigen, Gaithersburg, MD).
